# Inferring viral quasispecies spectra from 454 pyrosequencing reads

**DOI:** 10.1186/1471-2105-12-S6-S1

**Published:** 2011-07-28

**Authors:** Irina Astrovskaya, Bassam Tork, Serghei Mangul, Kelly Westbrooks, Ion Măndoiu, Peter Balfe, Alex Zelikovsky

**Affiliations:** 1 Department of Computer Science, Georgia State University, Atlanta, GA 30303, USA; 2 Life Technologies, Foster City, CA, USA; 3 Department of Computer Science & Engineering, University of Connecticut, Storrs, CT 06269, USA; 4 Institute of Biomedical Research, Birmingham University, Birmingham B15 2TT, UK

## Abstract

**Background:**

RNA viruses infecting a host usually exist as a set of closely related sequences, referred to as quasispecies. The genomic diversity of viral quasispecies is a subject of great interest, particularly for chronic infections, since it can lead to resistance to existing therapies. High-throughput sequencing is a promising approach to characterizing viral diversity, but unfortunately standard assembly software was originally designed for single genome assembly and cannot be used to simultaneously assemble and estimate the abundance of multiple closely related quasispecies sequences.

**Results:**

In this paper, we introduce a new **Vi**ral **Sp**ectrum **A**ssembler (ViSpA) method for quasispecies spectrum reconstruction and compare it with the state-of-the-art ShoRAH tool on both simulated and real 454 pyrosequencing shotgun reads from HCV and HIV quasispecies. Experimental results show that ViSpA outperforms ShoRAH on simulated error-free reads, correctly assembling 10 out of 10 quasispecies and 29 sequences out of 40 quasispecies. While ShoRAH has a significant advantage over ViSpA on reads simulated with sequencing errors due to its advanced error correction algorithm, ViSpA is better at assembling the simulated reads after they have been corrected by ShoRAH. ViSpA also outperforms ShoRAH on real 454 reads. Indeed, 7 most frequent sequences reconstructed by ViSpA from a real HCV dataset are viable (do not contain internal stop codons), and the most frequent sequence was within 1% of the actual open reading frame obtained by cloning and Sanger sequencing. In contrast, only one of the sequences reconstructed by ShoRAH is viable. On a real HIV dataset, ShoRAH correctly inferred only 2 quasispecies sequences with at most 4 mismatches whereas ViSpA correctly reconstructed 5 quasispecies with at most 2 mismatches, and 2 out of 5 sequences were inferred without any mismatches. ViSpA source code is available at http://alla.cs.gsu.edu/~software/VISPA/vispa.html.

**Conclusions:**

ViSpA enables accurate viral quasispecies spectrum reconstruction from 454 pyrosequencing reads. We are currently exploring extensions applicable to the analysis of high-throughput sequencing data from bacterial metagenomic samples and ecological samples of eukaryote populations.

## Background

### Viral quasispecies

Many viruses (including SARS, influenza, HBV, HCV, and HIV) encode their genome in RNA rather than DNA. Unlike DNA viruses, RNA viruses lack the ability to detect and repair mistakes during replication [1] and, as a result, their mutation rate can be as high as 1 mutation per each 1,000-100,000 bases copied per replication cycle [2]. Many of the mutations are well tolerated and passed down to descendants, producing a family of co-existing related variants of the original viral genome referred to as *quasispecies*, a concept that originally described a mutation-selection balance [3-7].

The diversity of viral sequences in an infected individual can cause the failure of vaccines and virus resistance to existing drug therapies [8]. Therefore, there is a great interest in reconstructing genomic diversity of viral quasispecies. Knowing sequences of the most virulent variants can help to design effective drugs [9,10] and vaccines [11,12] targeting particular viral variants *in vivo*.

### 454 pyrosequencing technology

Briefly, the 454 pyrosequencing system shears the source genetic material into fragments of approximately 300-800 bases. Millions of single-stranded fragments are sequenced by synthesizing their complementary strands. Repeatedly, nucleotide reagents are flown over the fragments, one nucleotide (A, C, T, or G) at a time. Light is emitted at a fragment location when the flown nucleotide base complements the first unpaired base of the fragment [13,14]. Multiple identical nucleotides may be incorporated in a single cycle, in which case the light intensity corresponds to the number of incorporated bases. However, since the number of incorporated bases (referred to as a homopolymer length) cannot be estimated accurately for long homopolymers, it results in a relatively high percentage of insertion and deletion sequencing errors (which respectively represent 65%-75% and 20%-30% of all sequencing errors [15,16]).

The software provided by instrument manufacturers were originally designed to assemble all reads into a single genome sequence, and cannot be used for reconstructing quasispecies sequences. Thus, in this paper we address the following problem:

### Quasispecies Spectrum Reconstruction (QSR) problem

Given a collection of 454 pyrosequencing reads generated from a viral sample, reconstruct the quasispecies spectrum, i.e., the set of sequences and the relative frequency of each sequence in the sample population.

A major challenge in solving the QSR problem is that the quasispecies sequences are only slightly different from each other. The amount and distribution along the genome of differences between quasispecies varies significantly between virus species, as different species have different mutation rates and genomic architectures. In particular, due to the lower mutation rate and longer conserved regions, HCV quasispecies are harder to reconstruct than quasispecies of HBV and HIV. Additionally, the QSR problem is made difficult by the limited read length and relatively high error rate of high throughput sequencing data generated by current technologies.

### Related work

The QSR problem is related to several well-studied problems: *de novo* genome assembly [17-19], haplotype assembly [20,21], population phasing [22] and metagenomics [23]. As noted above, *de novo* assembly methods are designed to reconstruct a single genome sequence, and are not well-suited for reconstructing a large number of closely related quasispecies sequences. Haplotype assembly does seek to reconstruct two closely related haplotype sequences, but existing methods do not easily extend to the reconstruction of a large (and *a priori* unknown) number of sequences. Computational methods developed for population phasing deal with large numbers of haplotypes, but rely on the availability of genotype data that conflates information about pairs of haplotypes. Metagenomic samples do consist of sequencing reads generated from the genomes of a large number of species. However, differences between the genomes of these species are considerably larger than those between viral quasispecies. Furthermore, existing tools for metagenomic data analysis focus on species identification, as reconstruction of complete genomic sequences would require much higher sequencing depth than that typically provided by current metagenomic datasets.

In contrast, achieving high sequencing depth for viral samples is very inexpensive, owing to the short length of viral genomes. Mapping based approaches to QSR are naturally preferred to *de novo* assembly since reference genomes are available (or easy to obtain) for viruses of interest, and viral genomes do not contain repeats. Thus, it is not surprising that such approaches were adopted in the two pioneering works on the QSR problem [24,25]. Eriksson et al. [24] proposed a multi-step approach consisting of sequencing error correction via clustering, haplotype reconstruction via chain decomposition, and haplotype frequency estimation via expectation-maximization, with validation on HIV data. In Westbrooks et al. [25], the focus is on haplotype reconstruction via transitive reduction, overlap probability estimation and network flows, with application to simulated error-free HCV data. Recently, the QSR software tool ShoRAH was developed [26] and applied to HIV data [27]. Another combinatorial method for QSR was also developed and applied to HIV and HBV data in [28], with results similar to those of ShoRAH. Our contributions in this paper are as follows:

• A novel QSR tool called **V**iral **S**pectrum **A**ssembler (ViSpA) taking into account sequencing errors at multiple steps,

• Comparison of ViSpA with ShoRAH on HCV synthetic data both with and without sequencing errors, and

• Statistical and experimental validation of the two methods on real 454 pyrosequencing reads from HCV and HIV samples.

## Methods

**Figure 1:**
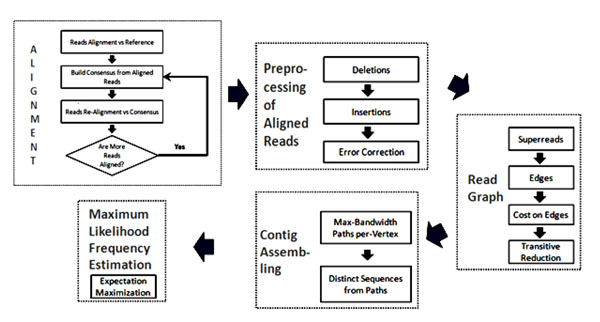
ViSpA’s flowchart.

Our method for inferring the quasispecies spectrum of a virus sample from 454 pyrosequencing reads consists of the following steps (see Fig. 1):

• Constructing the consensus virus genome sequence for the given sample and aligning the reads onto this consensus,

• Preprocessing aligned reads to correct sequencing errors,

• Constructing a transitively reduced read graph with vertices representing reads and edges representing overlaps between them,

• Selecting paths in the read graph that correspond to the most probable quasispecies sequences, and assembling candidate sequences for selected paths by weighted consensus of reads, and

• Estimating candidate sequence frequencies by EM

Below we describe each step separately.

### Read alignment and consensus genome sequence construction

We assume that a reference genome sequence of the particular virus strain is available (e.g., from NCBI [29]). Since viral genomes do not have sizable repeats and the quasispecies sequences are usually close enough to the reference sequence, the majority of reads can typically be uniquely aligned onto the reference genome. However, a significant number of reads may remain unaligned due to differences between the reference genome and sequences in the viral sample. In order to recover as many of these reads as possible, we iteratively construct a consensus genome sequence from aligned reads.

In particular, we first align 454 pyrosequencing reads to the reference sequence using the SEGEMEHL software [30]. Then we extend the reference sequence with a placeholder *I* for each nucleotide inserted by at least one uniquely aligned read. Similarly, we add a placeholder *D* to the read sequence for each reference nucleotide missing from the aligned read. Then we perform sequential multiple alignment of the previously aligned reads against this extended reference sequence. Finally, the consensus genome sequence is obtained by (1) replacing each nucleotide in the extended reference with the nucleotide or placeholder in the majority of the aligned reads and (2) removing all *I* and *D* placeholders, respectively corresponding to rare insertions and to deletions found in a majority of reads. Reads may contain a small portion of unidentified nucleotides denoted by *N*’s – we treat N as a special allele value matching any of nucleotides *A*, *C*, *T*, *G*, as well as placeholders *I*, and *D*.

Iteratively, we replace the reference with the consensus and try to align the reads, for which we could not find any acceptable alignment previously. Our experiments on a dataset consisting of approximately 31,000 454 pyrosequencing reads generated from a 5.2kb-long HCV fragment (see data description in Results and Discussions) show that 85% of reads are uniquely aligned onto the reference sequence and an additional 9% of the reads are aligned onto the final consensus sequence. Reads that cannot be aligned onto the final consensus are removed from the further consideration.

### Preprocessing of aligned reads

Since aligned reads contain insertions and deletions, we use placeholders *I* and *D* to simplify position referencing among the reads. All placeholders are treated as additional allele values but they are removed from the final assembled sequences. First, we substitute each deletion in the aligned reads with placeholder *D*. Deletion supported by a single read is replaced either with the allele value, which is present in all other reads overlapping this position, or with *N*, signifying an unknown value, otherwise. Next, we fill with placeholder *I* each gap in a read corresponding to the insertions in the other reads. All insertions supported by a single read are removed from consideration.

### Read graph construction

We begin with the definition of the read graph, introduced in [25] and independently in [24], and then describe the adjustments that need to be made to read graph construction and edge weights to account for sequencing errors as well as the high mutation rate between quasispecies.

The read graph *G* = (*V*, *E*) is a directed graph with vertices corresponding to reads aligned with the consensus sequence. For a read *u*, we denote by *b*(*u*), respectively *e*(*u*), the genomic coordinate at which the first, respectively the last, base of *u* gets aligned. A directed edge (*u*, *v*) connects read *u* to read *v* if a suffix of *u* overlaps with a prefix of *v* and they coincide across the overlap. Two auxiliary vertices - a source *s* and a sink *t* – are added such that *s* has edges into all reads with zero indegree and *t* has edges from all reads with zero outdegree. Then each *s* – *t*-path corresponds to a possible candidate quasispecies sequence. The read graph is transitively reduced, i.e., each edge *e* = (*u*, *v*) is removed if there is a *u* – *v*-path not including edge *e*. Note that certain reads can be completely contained inside other reads. Let a *superread* refer to a read that is not contained in any other read and let the rest of the reads be called *subreads.* Subreads are not used in the construction of the read graph, but are taken into account in the final assembly of candidate sequences and frequency estimation.

Since the number of different *s* – *t*-paths is exponential, we wish to generate a set of paths that have high probability to correspond to real quasispecies sequences. In order to estimate path probability, we independently estimate for each edge *e* the probability *p*(*e*) that it connects two reads from the same quasispecies, and then multiply estimated probabilities for all edges on the path. Under the assumption of independence between edges, if we assign to each edge *e* a cost equal to – log(*p*(*e*)) = log(1/*p*(e)), then the minimum-cost *s* – *t*-path will have the maximum probability to represent a quasispecies sequence.

For reads without errors, [25] estimated the probability that two reads *u* and *v* connected by edge (*u*, *v*) belong to the same quasispecies as(1)

where Δ = *b*(*v*) – *b*(*u*) is the *overhang* between reads *u* and *v*[25], *N* = #reads, *q* = #quasispecies, and *L* = *#*starting positions. Thus, in this case the cost of an edge with overhang Δ can be approximated by Δ ∝ log(1/*p*_Δ_).

To account for sequencing errors, we adjust the construction of the read graph to allow for mismatches. We use three parameters: (1) *n* = *#*mismatches allowed between a read and a superread, (2) *m* = #mismatches allowed in the overlap between two adjacent reads, and (3) *t* = *#*mismatches expected between a read and a random quasispecies. The probability that two reads *u* and *v* with *j* mismatches within an overlap of length *o* = *e*(*u*) – *b*(*v*) belong to the same quasispecies can be estimated as:(2)

where *ε* is the estimated 454 sequencing error rate. As in the case of error-free reads, defining the edge costs as  ensures that *s* – *t*-paths with low cost correspond to most likely quasispecies sequences.

### Candidate path selection

To generate a set of high-probability (low-cost) paths that are rich enough to explain observed reads, we compute for each vertex in the read graph the minimum cost *s* – *t*-path passing through it. Finding these paths is computationally fast. Indeed, we only need to compute two shortest-paths trees in G, one outgoing from *s* and one incoming into *t*; the shortest *s* – *t*-path passing through a vertex *v* is the concatenation of the shortest *s* – *v-*and *v* – *t*-paths.

Preliminary simulation experiments (see Additional File 1) show that better candidate sets are generated when edge costs c defined by (1) and (2) are replaced by *e^c^*. In fact, if we use even faster dependency on c then we obtain better candidate sets. The fastest growing cost effectively changes the shortest path into so called max-bandwidth path, i.e., paths that minimizes maximum edge cost for the entire path and for each subpath. So, ViSpA generates candidate paths using this strategy.

### Candidate sequence assembly

When no mismatches are allowed in the construction of the read graph, finding the candidate sequence corresponding to a *s* – *t*-path is trivial, since by definition adjacent superreads coincide across their overlap. When mismatches are allowed, we first assemble a consensus sequence from superreads used by the *s* – *t*-path. It may be not the best choice, especially when the coverage with superreads is low. Hence, we replace each initial candidate sequence with a weighted consensus sequence obtained using both superreads and subreads of the path, as described below.

For each read *r*, we compute the probability that it belongs to a particular initial candidate sequence *s* as:(3)

where l and *L* denote the lengths of the read and initial candidate sequence, respectively, *k* is the number of mismatches between the read and the initial candidate sequence *s*, and *t*/*L* is the estimated mutation rate. Then final candidate sequence is computed as the weighted consensus over all reads, where the weight of a read is the probability that it belongs to the sequence. Note that, unlike the case without mismatches, the same candidate sequence can be obtained from different candidate *s* – *t*-paths, so we remove duplicates at the end of this step.

### Estimation of candidate quasispecies sequence frequencies

We assume that reads *R* with observed frequencies  where generated from a quasispecies population *Q* as follows. First, a quasispecies sequence *q* ∈ *Q* is randomly chosen accordingly to its unknown frequency *f_q_.* A read starting position is generated from the uniform distribution and then a read *r* is produced from quasispecies *q* with *j* sequencing errors. The probability of this event is calculated as , where *l* is the read length and *ε* is the sequencing error rate. Thus, the probability of observing the read *r* under this model is .

Quasispecies frequencies are estimated by maximizing the log-likelihood function:

 using an EM algorithm [31] (see Additional File 1 for details). Currently, convergence of the EM algorithm is determined at the tolerance level 0.005.

## Results and discussions

In our simulation studies we use the following read data sets.

### Reads simulated from known HCV quasispecies

In order to perform cross-validation on the assembly method, we simulate reads data from 1739-bp long fragment from the E1E2 region of 44 HCV sequences [32] when sequence frequencies are generated according to some specific distribution. In our simulation experiments, we use geometric distribution (i-th sequence is constant factor more frequent than the (i + 1)-th sequence) to create sample quasispecies populations with different number of randomly selected above-mentioned quasispecies sequences.

We first simulate reads without sequencing errors: the length of a read follows normal distribution with a particular mean value and variance 400, and a starting position follows the uniform distribution. This simplified model of reads generation has two parameters: number of the reads that varies from 20K up to 100K and the average read length that varies from 200bp up to 600bp.

Additionally, we simulate 454 pyrosequencing reads from 10 quasispecies sequences (following geometric distribution of frequencies) out of 44 HCV sequences [32] using FlowSim [33]. We generated 30K reads with average length 350bp.

### 454 pyrosequencing reads from HCV samples

The data set Data1 has been received from HCV Research Group in Institute of Biomedical Research, at University of Birmingham. Data1 contains 30,927 reads obtained from the 5.2kb-long fragment of HCV-1a genome (which is more than a half of the entire HCV genome). The average (aligned) read length average is 292bp but it significantly varies as well as the depth of position coverage (see Additional File 1 for details). The depth of reads coverage variability is due to a strong bias in the sequence start points, reflecting the secondary structure of the template DNA or RNA used to generate the initial PCR products. As a result, shorter reads are produced by GC-rich sequences. Data1 is available upon request from the authors.

### 454 pyrosequencing reads from HIV samples

The HIV dataset [27] contains 55,611 reads from mixture of 10 different 1.5kb-long region of HIV-1 quasispecies, including *pol* protease and part of the *pol* reverse transcriptase. The aligned reads length varies from 35bp to 584bp with average about 345bp (see Additional File 1 for details). In contrast to [27], we do not filter out reads with low-quality scores.

### Experimental validation on simulated data

**Figure 2:**
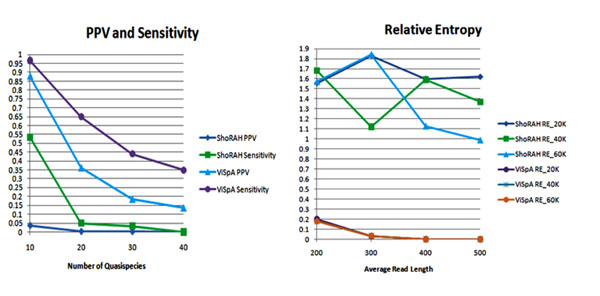
**Statistical validation on error-free reads from known HCV quasispecies.** Left: PPV and sensitivity as a function of the number of quasispecies in the original population (40K reads with average read length 300). Right: the relative entropy as a function of the average read length (40K reads from 10 quasispecies).

In all our experimental validations, we compare the proposed algorithm ViSpA with the state-of-the-art tool ShoRAH as well as with ViSpA on ShoRAH-corrected reads (ShoRAHreads + ViSpA). We say the quasispecies sequence is captured if one of the candidate sequences exactly matches it. We measure the quality of assembling by portion of the real quasispecies sequences being captured by candidate sequences  and its portion among candidate sequences (positive predictive value ) in cross-validation tests. Both sensitivity and PPV are analyzed as functions of the number of quasispecies in underlying sample population (see Fig. 2 (left)). ViSpA can correctly assemble all sequences out of 10 quasispecies and 29 sequences out of 40 quasispecies if average read length is at least 300bp. If the average read length is smaller (for example, in range from 250bp till 299bp), the method can assemble at least 8 out of 10 sequences and 20 out of 40 sequences. Here, we see advantage of ViSpA over ShoRAH.

Following [24], we measure the prediction quality of frequency distribution with Kullback-Leibler divergence, or relative entropy. Given two probability distributions, relative entropy measures the ”distance” between them, or, in the other words, the quality of approximation of one probability distribution by the other distribution. Formally, the relative entropy between true distribution *P* and approximation distribution *Q* is given by the formula:

where summation is over all reconstructed original sequences *I* = {*i* | *P*(*i*) > 0, *Q*(*i*) > 0} , i. e., over all original sequences that have a match (exact or with at most *k* mismatches) among assembled sequences. The relative entropy is decreasing with increasing of the average read length. It is expected since sensitivity is increasing with increasing of the average read length and EM predicts underlying distribution more accurately. ViSpA algorithm considerably outperforms ShoRAH (see Fig. 2 (right)).

However, ShoRAH has a significant advantage over ViSpA on a read data simulated by FlowSim both in prediction power and in robustness of results (see Table 1). Indeed, ShoRAH correctly infers 3 out of 10 real quasispecies sequences whereas ViSpA reconstructs only 1 sequence. Additionally, 10 most frequent assemblies inferred by ShoRAH are more robust with repeating up to 45% of times on 10%-reduced data versus 1% of times for ViSpA’s assemblies. This advantage can be explained by superior read correction in ShoRAH. If ViSPA is used on ShoRAH-corrected reads, the results drastically improves: 5 quasispecies sequences are inferred and exactly 95% of times are repeated on reduced data, confirming that ViSpA is better in assembling sequences (see Table 1).

**Table 1:** Comparison of three methods – ViSpA, ShoRAH, and ShoRAHreads+ViSpA – on the read data simulated by FlowSim.

	ShoRAH				ViSpA				ShoRAHreads+ViSpA			
	
	PPV	Sensitivity	Reproducibility		PPV	Sensitivity	Reproducibility		PPV	Sensitivity	Reproducibility	
									
			Max	Average			Max	Average			Max	Average
k=0	0.0097	0.3	0.45	0.11	0.0008	0.1	0.1	0.1	0.5	0.5	0.95	0.95
k=1	0.0129	0.4	0.6	0.32	0.0008	0.1	0.1	0.1	0.5	0.5	0.95	0.95
k=9	0.0162	0.5	0.95	0.64	0.0015	0.2	0.1	0.1	0.5	1	0.95	0.95

The quasispecies sequence is considered found if one of candidate sequences matches it exactly (*k* = 0) or with at most *k* (1 or 9) mismatches. All methods are run 100 times on 10% - reduced data. For the *i*-th (*i* = 1, .., 10) most frequent sequence assembled on the whole data, we record its reproducibility, i.e., percentage of runs when there is a match (exact or with at most *k* mismatches) among 10 most frequent sequences found on reduced data. ”Reproducibility: Max” and ”Reproducibility: Average” report respectively maximum and average of those percentages.”

### Experimental validation on 454 pyrosequencing reads from HCV samples

We first discuss the choice of parameters of the read graph and candidate sequence assembly from s – *t*-paths. Then we give statistical validation for obtained 10 most frequent quasispecies sequences.

**Figure 3:**
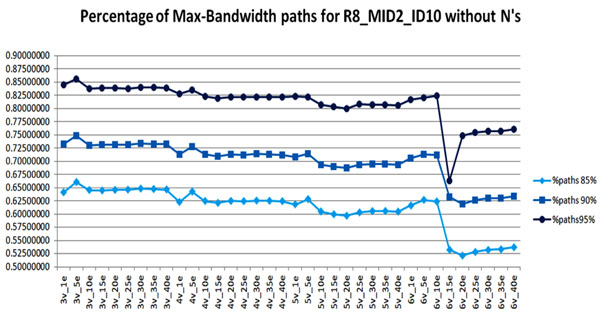
**Percentage of candidate sequences which cumulative frequency is 85%, 90%, and 95%.** The values on x-axis corresponds to the number of allowed mismatches during read graph construction. *n_m* means that up to *n* mismatches are allowed in superreads and up to *m* mismatches are allowed in edges.

We infer quasispecies spectrum based on the read graphs constructed with various numbers *n* and *m* (numbers of mismatches allowed for superreads and overlaps corresponding to edges). We sort the estimated frequencies in descending order and count the number of sequences which cumulative frequency is 85%, 90%, and 95%. Fig. 3 reports these numbers as a percent of the total number of candidate sequences. There is an obvious drop in percentage for all three categories if we allow up to *n* = 6 mismatches to cluster reads and up to *m* = 15 mismatches to create edges. In this case, the constructed read graph has no isolated vertices.

To refine assembled candidate sequences, we use all reads and parameter *t* varying from 80bp till 350bp, or, in the other words, mutation rate varying from 1.75% up to 8% per sequence (which is in the range observed in [34]). Out of 3207 max-bandwidth paths, we obtain as much as 938 distinct sequences (*t* = 80) and as low as 755 sequences (*t* = 350) for different values of *t* ∈ [80; 350].

**Figure 4:**
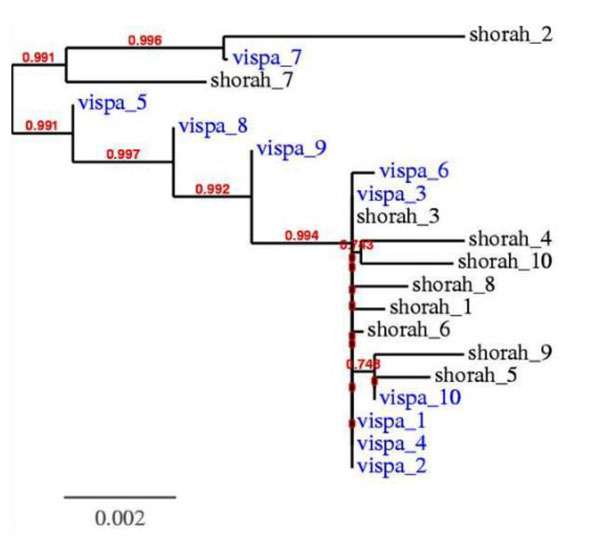
**The neighbor-joining phylogenetic tree for 10 most frequent HCV quasispecies variants on a 5,205bp-long fragment obtained by ViSpA and ShoRAH.** Sequences are labeled with software name and its rank among 10 most frequent assembled sequences.

The neighbor-joining tree for the most frequent 10 candidate sequences obtained by ViSpA and ShoRAH (see Fig. 4) reminds a neighbor-joining tree for HCV quasispecies evolution. Additionally, the most frequent candidate sequence found by ViSpA is 99% identical to one of the actual ORFs obtained by cloning the quasispecies.

Viral sequences containing internal stop codons are not viable since the entire HCV genome consists of a single coding region for a large polyprotein. So the number of reconstructed viable sequences can serve as an accuracy measure for quasispecies assembly. Out of 10 most frequent sequences reconstructed by ViSpA, only 3 are not viable while ShoRAH is able to reconstruct only one viable sequence. This sequence has 99.94% similarity with the ViSpA’s fourth most frequent assemblies. Both methods returned similar frequency estimations for this sequence: 0.017% (ShoRAH) and 0.019% (ViSpA).

Both ShoRAH and ViSpA (*n* = 6, *m* = 15) are run on eight 2.66GHz-CPUs with 8M cache. They take around 40 minutes to assemble sequences and estimate their frequencies. Smaller value of *n* increases ViSpA’s runtime since its bottleneck (candidate sequences assembling) is proportional to the number of reads times number of paths. Indeed, smaller value of *n* results in larger number of superreads in built read graph, thus, in larger set of candidate paths. For example, ViSpA runs 90 minutes for *n* = 2, *m* = 2.

### Statistical validation of the quasispecies spectrum

**Figure 5:**
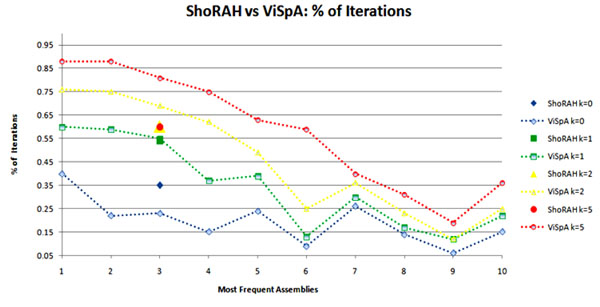
**Percentage of runs when the i-th most frequent sequence is reproduced among 10 most frequent quasispecies assembled on the 10%-reduced set of reads.** The *i*-th point at x-axis corresponds to the *i*-th most frequent sequence assembled on the 100% of reads. No data are shown for the sequences that are reproduced less than 5% of runs.

The plot on Fig. 5 shows validation results for 10 most frequent quasispecies sequences with respect to EM estimations assembled on Data1 by ShoRAH and ViSpA (*n* = 6, *m* = 15, and *t* = 120). Repeatedly, 100 times we have deleted randomly chosen 10% of reads and run both methods on each reduced read instance to reconstruct quasispecies spectrum.

The plot reports the percentage of runs when each of 10 most frequent sequences assembled on Data1 are reproduced among the 10 most frequent quasispecies inferred on the reduced instances with no mismatches (*k* = 0), or with *k* = 1, 2, 5 mismatches. For example, for *k* = 0 ShoRAH repeatedly (35% of times) reconstructs only the third most frequent sequence while ViSpA reconstructs 7 sequences in at least 15% times, and the most frequent sequence is reconstructed 40% times. This plot shows that the found sequences are pretty much reproducible for ViSpa.

### Experimental validation on 454 pyrosequencing reads from HIV samples

In order to compare ViSpA and ShoRAH, we run both of the methods on HIV dataset, used in the first experiment in [27]. As said above, we do not preprocess reads with respect to its 454 quality score, and it can explain poorer performance of ShoRAH. Indeed, ShoRAH correctly infers only 2 quasispecies sequences with at most 4 mismatches: one assembly has 3 mismatches with real quasispecies sequence, and the other has 4 mismatches.

ViSpA correctly reconstructs 5 quasispecies with at most 2 mismatches (3 of them among 10 most frequent assemblies): two sequences are inferred without any mismatches (one is among 10 most frequent assemblies), one assembly has 1 mismatch with real quasispecies sequence (and it is among 10 most frequent assemblies), and the rest sequences have 2 mismatches (one is among 10 most frequent assemblies). The assemblies correspond to a viable protein sequences.

If ViSpA is applied to ShoRAH-corrected reads, it can successfully infer three real quasispecies without any mismatches.

## Conclusions

In this paper, we have proposed and implemented ViSpA, a novel software tool for quasispecies spectrum reconstruction from high-throughput sequencing reads. The ViSpA assembler takes into account sequencing errors at multiple steps, including mapping-based read preprocessing, path selection based on maximum bandwidth, and candidate sequence assembly using probability-weighted consensus techniques. Sequencing errors are also taken into account in ViSpA’s EM-based estimation of quasispecies sequence frequencies.

We have validated our method on simulated error-free reads, FlowSim-simulated reads with sequencing errors, and real 454 pyrosequencing reads from HCV and HIV samples. We are currently exploring extensions of ViSpA to paired-end reads; the main difficulty is selection of pair-aware candidate paths. We also foresee application of ViSpA’s techniques to the analysis of high-throughput sequencing data from microbial communities [23] and ecological samples of eukaryote populations [35].

## Availability

The ViSpA source code is available at http://alla.cs.gsu.edu/~software/VISPA/vispa.html.

## Authors contributions

IA designed algorithms, developed software, performed analysis and experiments, wrote the paper. BT performed analysis and experiments. SM contributed to developing software. KW designed algorithms and developed software. IM contributed to designing the algorithms and writing the paper. PB supplied the HCV data and contributed to performing the analysis. AZ designed the algorithms, wrote the paper and supervised the project. All authors have read and approved the final manuscript.

## Competing interests

The authors declare that they have no competing interests.

## Supplementary Material

Additional file 1 **Supplementary Materials.** The file contains derivation of edge cost formula (2) and EM algorithm, example of read graph construction and analysis of 454 pyrosequencing data. Click here for file
