# Supplementary material for: Inferring viral quasispecies spectra from 454 pyrosequencing reads
Source: BMC Bioinformatics. 2011 Jul 28;12(Suppl 6):S1. doi: 10.1186/1471-2105-12-S6-S1 (PMC3194189; doi:10.1186/1471-2105-12-S6-S1)
Supplement: Additional file 1 — Supplementary Materials. The file contains derivation of edge cost formula (2) and EM algorithm, example of read graph construction and analysis of 454 pyrosequencing data. [file 1471-2105-12-S6-S1-S1.pdf]

Supplementary Materials
for
Inferring Viral Spectrum from 454 Pyrosequencing Reads

Irina Astrovskaya, Bassam Tork, Serghei Mangul, Kelly Westbrooks, Ion Mandoiu, Peter Balfe, and Alex Zelikovsky

1 Methods

Cost Formula Derivation. First, let us consider a simplified model where all quasispecies are uniformly distributed and reads beginning positions follow uniform distribution as well. Let $b(u)$ and $e(u)$ be the beginning and the end positions of the read u, respectively. And let A be an event that two reads u, v from the same quasispecies Q are connected with an edge (u, v) in the transitively reduced graph, and j differences among u and v occur in the overlap due to sequencing errors. In the other words, the event A is a product of the following independent events: (1) read u exists, (2) read v exists, starting at position $b(v) = b(u) + \Delta$ (overhang Δ is some shift) (3) no read w from the same quasispecies Q satisfies $b(u) < b(w) < b(v)$, and (4) there are j sequencing errors in overlap $o = e(u) - b(v)$ of reads u and v.

Given N reads, originated from q quasispecies of length L, the probability that read u starts at a position $b(u)$ is $\frac{N}{Lq}$. The probability of the event $\Delta > k$ is the probability that there is no read from quasispecies Q starting at any position in $\{b(u) + 1, b(u) + 2, \ldots, b(u) + k\}$, that is

$$p_k = \left(1 - \frac{N}{Lq}\right)^k \approx \exp(-kN/Lq).$$

Then

$$Pr(\Delta = k) = \left(\frac{N}{Lq}\right)^2 p_{k-1}.$$

Next we calculate the probability of having j genotyping errors among overlapped positions as follows:

$$p(j) = \binom{o}{j} (1 - \varepsilon)^{o-j} \varepsilon^j,$$

where ε is a genotyping error rate. Finally, the probability of event A is:

$$Pr(A) = p(\Delta_j) = Pr(\Delta = k)p(j) \approx \left(\frac{N}{Lq}\right)^2 \exp(-\Delta N/Lq) \binom{o}{j} (1 - \varepsilon)^{o-j} \varepsilon^j$$

where $\Delta = b(v) - b(u)$ is a shift (overhang) between starting positions of reads u and v.

If $\Delta \gg Lq/N$ then v is more likely to be a read from another quasispecies Q' and differences from quasispecies Q and Q' in interval between $b(u)$ and $b(v)$ are the cause of large Δ.

Therefore,

$$1/p_{\Delta_j} \approx \frac{\exp(\Delta N/Lq)}{\binom{o}{j} (1 - \varepsilon)^{o-j} \varepsilon^j} \quad (1)$$

measures our uncertainty that (u, v) is a true edge.

In practice, quasispecies frequencies are under some unknown distribution F. Since it is impossible to approximate F, we assume that two identical copies of the same quasispecies genome correspond to two different entities, which follow uniform distribution.

In experimental results, reads starting positions tend to follow uniform distribution with small amount of outliers. If it is not a case, the cost formula can be adjusted by taking into account number of reads starting in interval between $b(u)$ and $b(v)$. However, when a candidate path is constructed, at each step, algorithm chooses between edges spanning almost the same positions, thus, non-adjusting cost formula to the coverage does not influence the construction of a candidate path.

Frequency Estimation via EM-algorithm.

Once a set of candidate sequences is obtained, their maximum-likelihood frequencies are calculated by the EM based algorithm. Iteratively, we estimate missing probability $p_{q,r}$ that read r comes from a candidate sequence q with j sequencing errors and maximize likelihood of an approximated model.

First, we create a bipartite graph $G = \{Q \bigcup R, E\}$ such that each candidate sequence is represented as a vertex $q \in Q$, and each read is represented as a vertex $r \in R$. With each vertex $q \in Q$, we associate unknown frequency f_q of the candidate sequence. And with each vertex $r \in R$, we associate read observed frequency o_r. Then for each pair q, r, we add an edge (q, r) weighted by probability of the read r being produced by the candidate sequence q with j genotyping errors:

$$h_{q,r} = \binom{l}{j} (1 - \varepsilon)^{l-j} \varepsilon^j,$$

where l is length of read sequence, and ε is the genotyping error rate.

After initializing frequencies $f_{q_{q \in Q}}$ at random, the algorithm repeatedly performs the next two steps until convergence:

E-step: For each pair q, r, compute the expected value $p_{q,r}$ that read r comes from candidate sequence q under the assumption that frequencies $f_{q_{q \in Q}}$ are correct by the following formula:

$$p_{q,r} = \frac{f_q \cdot h_{q,r}}{\sum\limits_{q' : (q', r) \in E} f_{q'} \cdot h_{q',r}}.$$

M-step: For each $q \in Q$, update value of f_q to the portion of reads being originated by the candidate sequence q among all observed reads in the sample, i.e.:

$$f_q = \frac{\sum\limits_{r : (q,r) \in E} p_{q,r} \cdot o_r}{\sum\limits_{r \in R} o_r}.$$

Currently, convergence of EM algorithm is determined at the tolerance level 0.005.

Rationale for Max-Bandwidth Path. Previously, we show that cost of an edge should be correlated to its overhang (shift) Δ. If we view the costs on edges as edge lengths, then the most probable paths for quasispecies are the shortest paths in the graph. So we can choose shortest path for each vertex to build the set of candidate paths.

In experiments on error-free reads, we consider the family of edge cost functions $cost_k(u, v) = e^{\frac{\Delta(u,v)}{k}}$. Figure 1 shows the number of the shortest paths in candidate set as a function of k. Smaller values of k yield fewer paths, and surprisingly, no correct candidate quasispecies are lost with decreasing of k. In the limiting case $k = 0$, the resulted paths are maximum bandwidth paths.

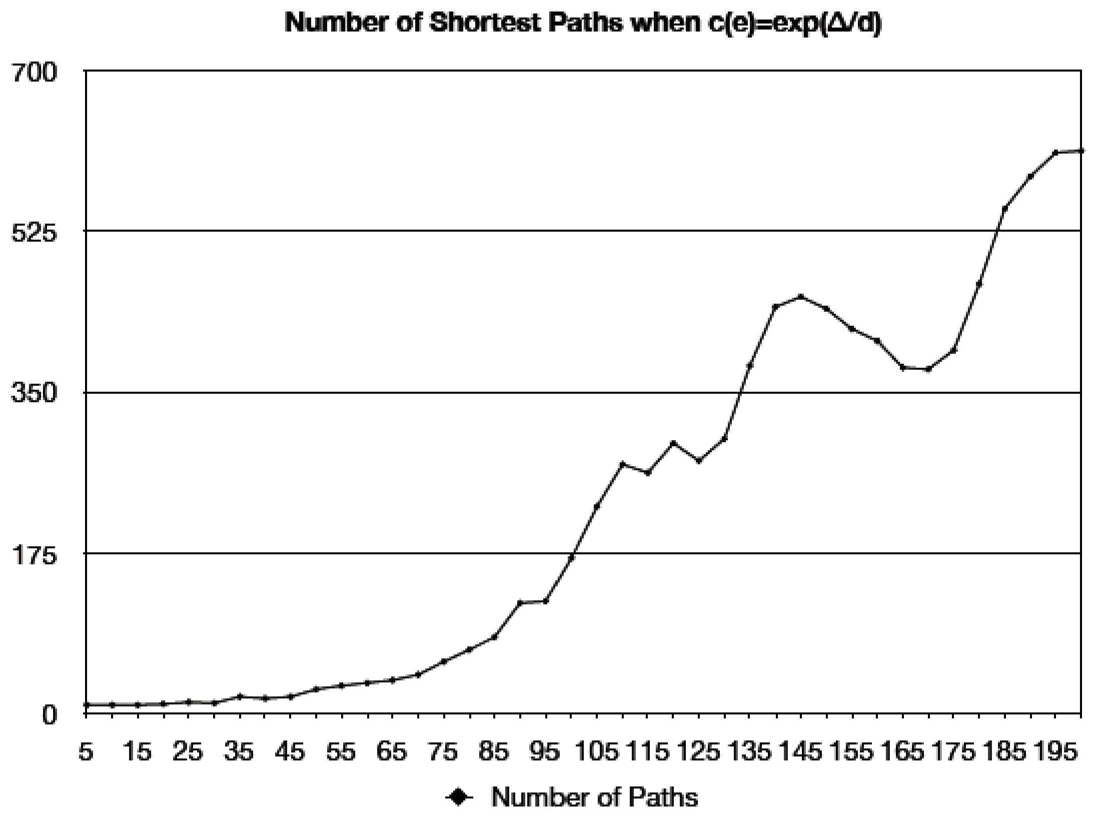

Fig. 1. The number of shortest paths in the read graph as a function of k when the edge cost function is $e^{\frac{\Delta}{k}}$.

Example of Read Graph Construction.

```
Reference: ...AGCGT---GAAGCT--T...
    Read1: ...AG-GTG--GAAGCT
    Read2: ...AGA-TGGAGAAA-T--T...
    Read3: ...AGCGA---GTCG-TAAT...
    Read4: ...AGCGA---GTCACT--T...

Reference: ...AGCGTIIIGAAGCTIIT...
    Read1: ...AGDGTGIIGAAGCT
    Read2: ...AGADTGGAGAAADTIIT...
    Read3: ...AGCGAIIIGTCGDTAAT...
    Read4: ...AGCGAIIIGTCACTIIT...

Reference: ...AGCGTIGAAGCTT...
    Read1: ...AGNGTGGAAGCT
    Read2: ...AGAGTGGAAADTT...
    Read3: ...AGCGAIGTCGDTT...
    Read4: ...AGCGAIGTCACTT...
```

Fig. 2. Example of indels preprocessing and simple error correction. At the top, multiple reads alignment is given. At the middle, I and D placeholders are added. At the bottom, deletions, supported by a single read, are corrected, insertions, confirmed by a single read, are removed.

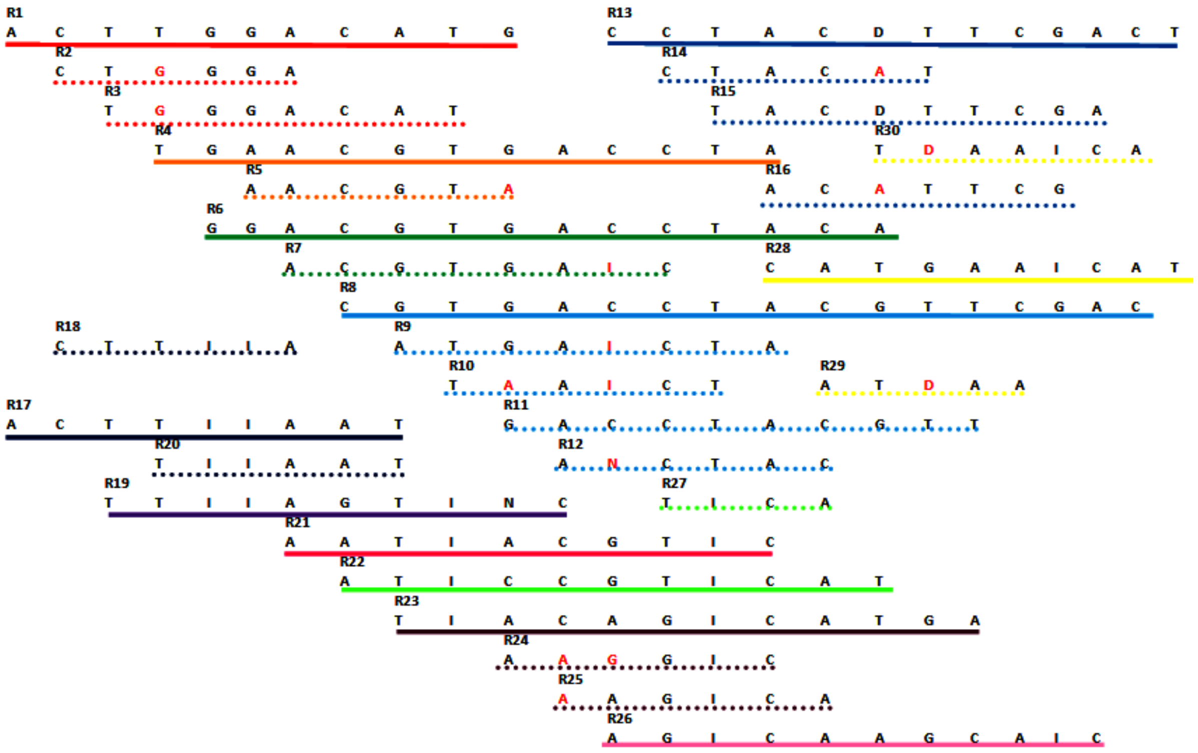

Fig. 3. Example of aligned reads. Reads are divided into superreads and subreads ($n = 2$). Reads underlined by bold lines are superreads. Reads underlined by dashed lines are subreads. Superread and its subreads are underlined by line of the same color. If subread has mismatch with its superread, the mismatch is colored in red. For example, $R2$ and $R3$ are subreads of $R1$ superread, $R2$ and $R3$ have only one difference with $R1$.

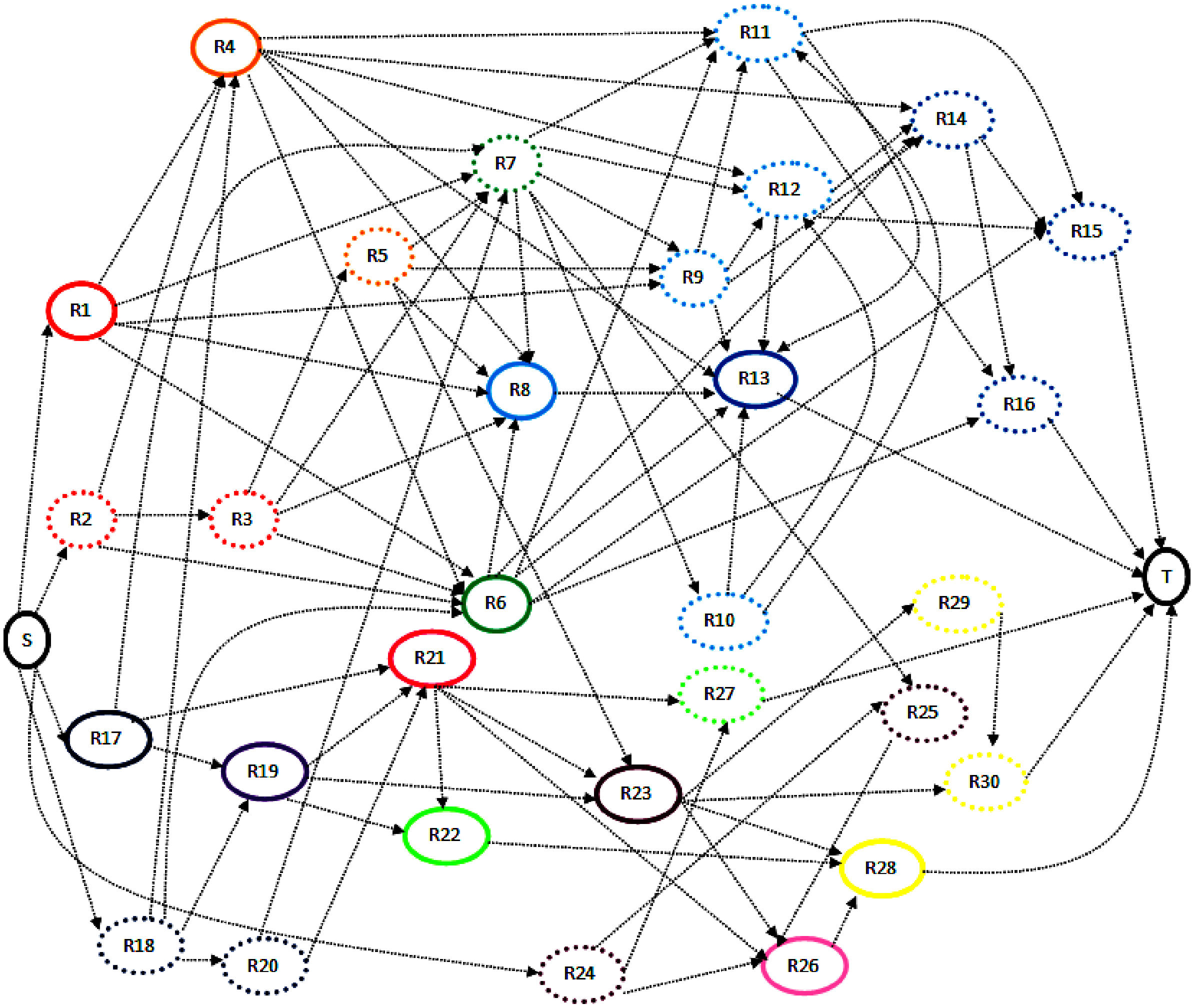

Fig. 4. Example of read graph ($n = 2, m = 2$) if both superreads and subreads are represented by vertices in the read graph. Vertices circled by bold lines correspond to superreads. Vertices circled by dashed lines correspond to subreads. Vertices that correspond to superread and its subreads are circled by the same color.

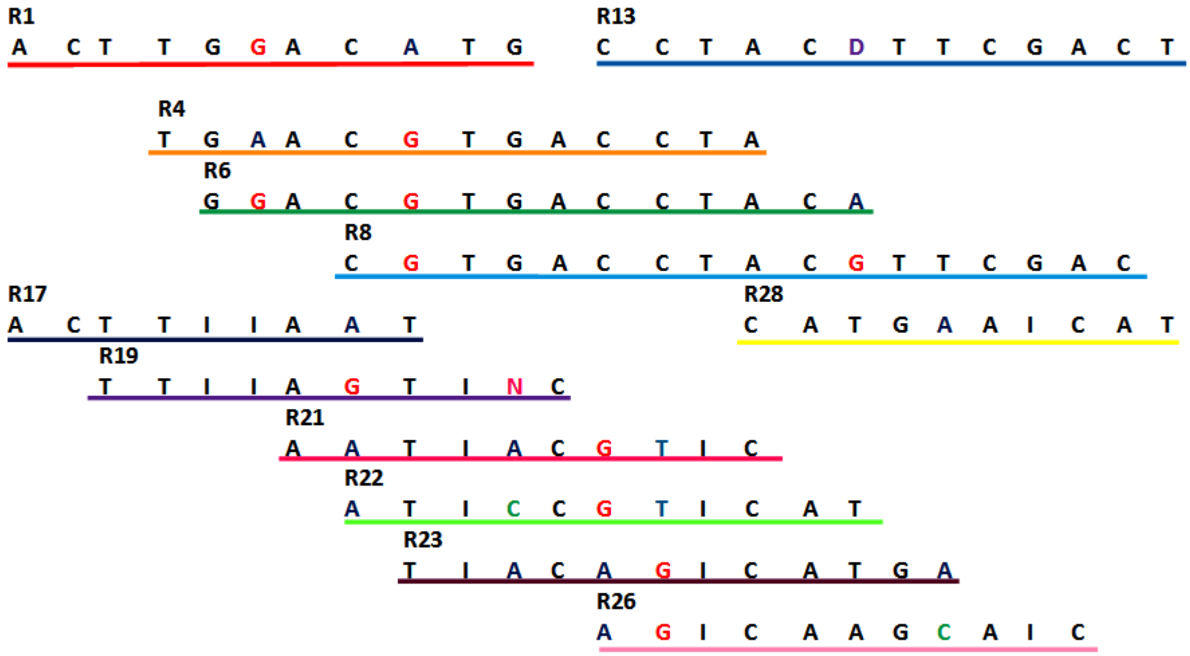

Fig. 5. Example of aligned superreads ($n = 2$). If there are several allelic values across reads in a position, they are marked by different colors to illustrate differences in overlap. Differences are marked across reads $R1 - R13$ and $R17 - R28$, separately. Colors are as follows: red is for "G", dark blue is for "A", green is for "C", blue is for "T", pink is for "N" and purple is for "D". For example, $R1$ and $R4$ have 2 differences in the overlap, $R1$ and $R6$ as well as $R1$ and $R6$ have 1 difference.

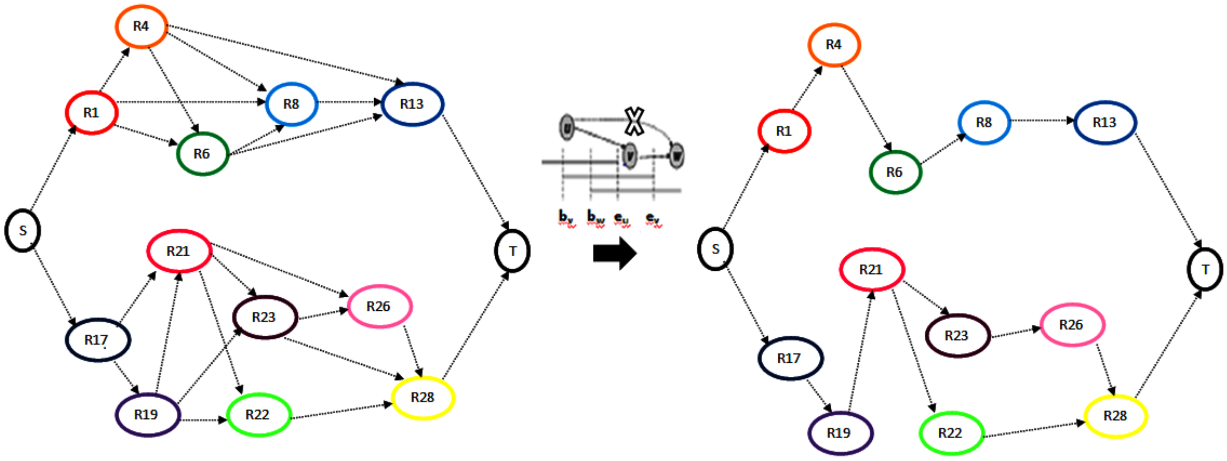

Fig. 6. Example of read graph ($n = 2, m = 2$) if only superreads are represented by vertices in the read graph. Read graph is shown before transitive reduction (at the left) and after transitive reduction was applied (at the right).

2 Data Sets

Reads generated by FlowSim from known HCV quasispecies.

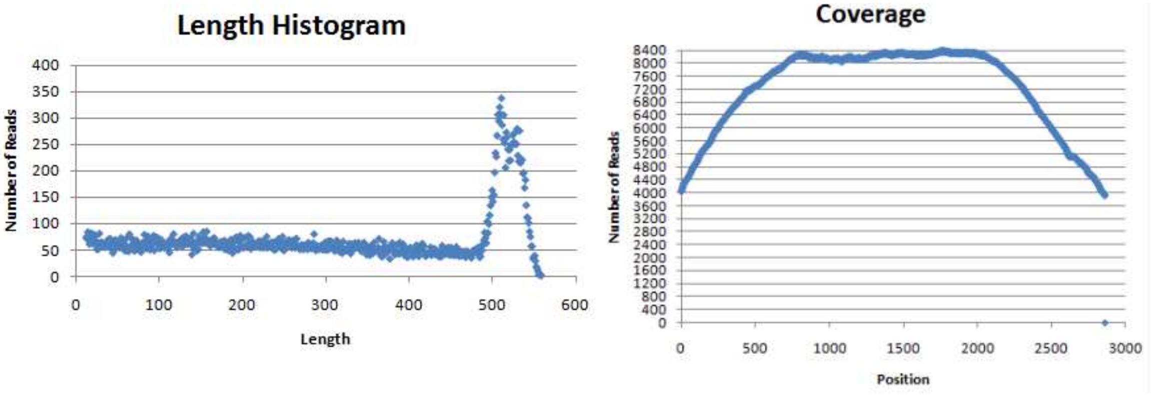

Fig. 7. Length histogram and position coverage for reads generated by FlowSim. Left: number of aligned reads with given length. Right: number of aligned reads covering extended reference positions. Each position is covered by at least 4000 reads, except the position at the very end.

Additional Read Statistics. 99.96% of aligned reads has at least one indel with respect to the reference: 99.97% of deletions and 99.6% of insertions are 1bp long. Only 1.1% of aligned reads has unknown value(s).

454 Pyrosequencing Reads from HCV Samples.

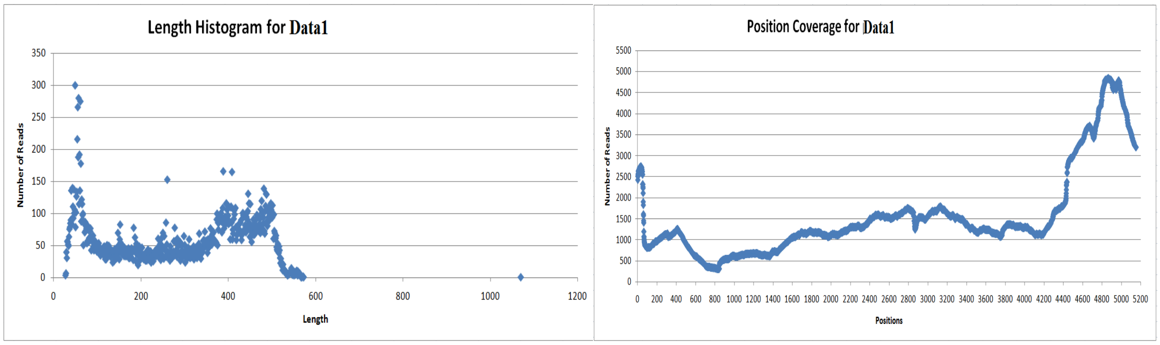

Fig. 8. Left: number of aligned reads with given length. There is a single read 1050bp long, major peak at 450bp long and 50bp long. Right: number of aligned reads covering extended reference positions. The global minimum is 800th nucleotide covered by 310 reads.

Additional Read Statistics. 72% of aligned reads has at least one deletion with respect to the reference: 98% of deletions are 1bp long, 1.5% has length 2, and the rest 0.5% has length 3. 77% of aligned reads has at least one insertion: 86% of insertions have length equaled to 1, and 9.8% have length equaled to 3. Only 7% of aligned

reads has at least one unknown value. Assuming that only once encountered insertions caused by typing errors, we found that the insertion error rate is at least 0.025%.

454 Pyrosequencing Reads from HIV Samples.

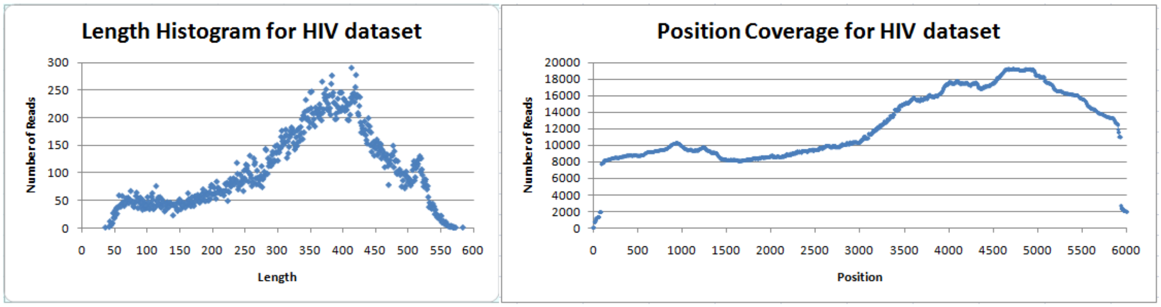

Fig. 9. Left: number of aligned reads with given length. Right: number of aligned reads covering extended reference positions.

Additional Read Statistics. 87% of aligned reads has at least one deletion with respect to the reference: 99.97% of deletions are 1bp long. 99% of aligned reads has at least one insertion: 85% of insertions have length equaled to 1, 10% have length equaled to 2, and 3.5% have length equaled to 3. 11.6% of aligned reads has at least one unknown value.
